# Maximizing wireless power transfer efficiency at exceptional points

**DOI:** 10.1038/s44172-025-00445-y

**Published:** 2025-06-10

**Authors:** Wei-Kang Hu, Bowang Zhang, Youhao Hu, Haoxiang Li, Wei Han

**Affiliations:** 1 Division of Emerging Interdisciplinary Areas, The Hong Kong University of Science and Technology, Hong Kong SAR, China; 2 Sustainable Energy and Environment Thrust, The Hong Kong University of Science and Technology (Guangzhou), Guangzhou, China; 3 Advanced Materials Thrust, The Hong Kong University of Science and Technology (Guangzhou), Guangzhou, China

**Keywords:** Electrical and electronic engineering, Phase transitions and critical phenomena

## Abstract

Magnetically coupled wireless power transfer (WPT) technology has been applied in stationary consumer electronics and holds great potential for charging traveling electric vehicles. The efficiency of WPT systems is, however, inherently vulnerable to changes in coupling and load conditions. Previous studies have underscored the robustness of parity-time (PT) symmetric WPT systems against variations in coupling strength within the exact PT region. In this study, by treating the loss rate as an adjustable parameter, we unveil that the efficiency of a PT-symmetric WPT system reaches its peak at the exceptional point (EP). Through adaptive adjustment of the virtual loss rate that pins the system at the EP, we can uphold maximum‐efficiency, frequency‐stable power transfer under a broad range of coupling and load conditions. Our EP-pinning strategy offers a significant advantage over conventional schemes that require on-site measurement of coupling strength and loss rates. The discovery of EP-induced efficient power transfer should facilitate the future deployment of wireless charging infrastructure.

## Abstract (web summary)

Wireless power transfer efficiency often drops when distance and load change. Wei-Kang Hu and colleagues show efficiency peaks at the exceptional point (EP) and propose an EP-pinning strategy to auto-adjust load loss for maximum efficiency.

## Introduction

Wireless power transfer (WPT) is increasingly applied in consumer electronics^1,2^, sensors^3^, medical devices^4^, and even flexible microsystems and flapping-wing aerial vehicles^5,6^. Embedding power transmission pads into roadways enables dynamic wireless charging^7,8^, potentially easing range anxiety and reducing battery costs for electric vehicles^9^. In a typical WPT system^10^, a transmitter converts electrical energy into an intermediate form (magnetic, electric, electromagnetic, or acoustic), which is then transmitted through air and reconverted into electrical energy by the receiver^11,12^. Magnetic fields excel at near-field WPT^13–16^, but magnetically coupled systems face one major challenge^17^: transfer efficiency instability due to variations in coupling strength and loss rate.

Parity-time (PT) symmetric systems continuously exchange energy with their surroundings while maintaining balanced input and output to conserve energy^18^. A non-Hermitian Hamiltonian meeting PT-symmetry conditions can exhibit purely real eigenvalues, with modal transitions triggered by parameter changes^19–22^. At the exceptional point (EP), these splitting eigenmodes merge into a degenerate state^23,24^. Given the structural similarities between the Schrödinger equation and the electromagnetic wave equation, PT-symmetric modes were widely used in optics and electronics, leading to breakthroughs in single-mode lasers^25,26^, hardware encryption^27^, and switching oscillation suppression^28^. EP‐focused optimization has been reported for realizing sensitivity enhancement^29,30^ and optimal switching in photonics^31^. The incorporation of PT symmetry into the design of robust WPT systems against variable coupling strength marks a significant advancement^32,33^. The operational amplifier-based nonlinear gain element, used in the initial design of a robust PT-symmetric WPT system^34^, was improved with a class-E power amplifier to enhance energy injection efficiency^35^. Heterogeneously coupled neutrals, configured in PT symmetry, were added to enlarge the robust region of WPT^36^.

Prior investigations into PT-symmetric WPT have predominantly focused on coupling strength as the variable parameter, utilizing the invariance of reflective impedance within the exact PT region to develop a robust system impervious to coil displacement^37^. By treating the loss rate as the main tuning parameter, in this study, we unveil the hidden correlation between the WPT efficiency and the EP of the PT symmetry transition. We demonstrate an EP-pinning strategy that can maintain the maximum efficiency and frequency-stable operation of a PT-symmetric WPT system. This EP-centered control strategy obviates the need for measuring dispersed circuit parameters such as coupling strength and loss rates in conventional schemes for maximum efficiency tracking^38,39^, as these are inherently embodied in the frequency generated by the nonlinear gain.

## Results

### Exceptional point and maximum efficiency

**Fig. 1:**
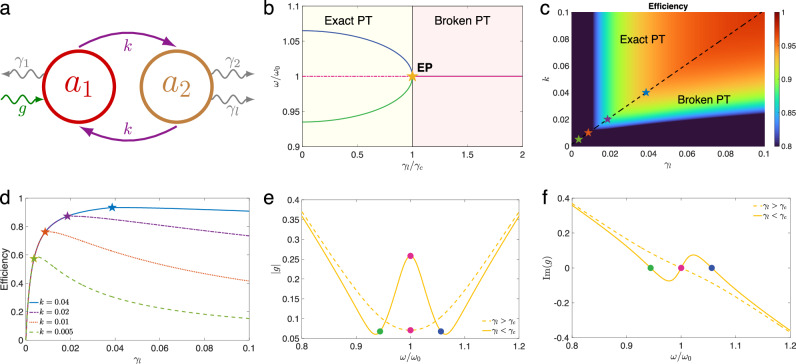
Phase diagram and EPs of a PT-symmetric WPT system. **a** A schematic representation of a generic non-Hermitian system composed of two coupled entities with gain and losses. **b** Frequency spectrum of a two-level PT-symmetric system with respect to load loss. **c** Transfer efficiency as a function of coupling strength and load loss in a nonlinear PT-symmetric WPT system; the dashed black line lies on the perfect matched condition where EPs emerge, and efficiency reaches maximum. **d** A slice of (**c**) given a set of coupling strengths. **e**, **f** Underlying phase transition diagrams illustrating the modulus (**e**) and imaginary part (**f**) of gain as functions of operating frequency. The colored points represent three eigenfrequencies (blue, green, and red for high, low, and natural resonant frequency).

A PT-symmetric WPT system is characterized by the interplay between the gain and loss of two coupled resonators (Fig. 1a). Energy flows into the transmitter resonator ${a}_{1}$ with the gain rate $g$ and dissipates with the loss rate ${\gamma }_{1}$. The receiver resonator ${a}_{2}$ is purely lossy, with the intrinsic loss ${\gamma }_{2}$ and load loss ${\gamma }_{l}$. The coupling $k$ between the two resonators acts as a conduit for power transfer from the transmitter to the receiver, culminating in power delivery to the load. The temporal evolution of the coupled-mode amplitudes of the transmitter and receiver resonators is governed by a set of coupled differential equations reflecting the dynamics of PT-symmetric systems. Specifically, the mathematical representation can be expressed as follows^34,35^:

1 $$i\frac{d}{{dt}}\left[\begin{array}{c}{a}_{1}\\ {a}_{2}\end{array}\right]=\frac{{\omega }_{0}}{2}\left[\begin{array}{cc}2+i\left(g-{\gamma }_{1}\right) & k\\ k & 2-i\left({\gamma }_{2}+{\gamma }_{l}\right)\end{array}\right]\left[\begin{array}{c}{a}_{1}\\ {a}_{2}\end{array}\right]$$

where ${a}_{1}$ and ${a}_{2}$ denote the mode amplitudes of the transmitter and receiver resonators, respectively; ${\omega }_{0}$ represents the natural resonant frequency of the two resonators and $i$ is the imaginary unit. In a nonlinear PT-symmetric WPT system, the value of $g$ is determined by other modal parameters, which can be derived by solving $\det \left(H-\omega I\right)=0$, where $H$ is the Hamiltonian matrix and $I$ represents the identity matrix:

2 $$g={\gamma }_{1}+2\frac{{\omega }_{0}-\omega }{{\omega }_{0}}i+\frac{{k}^{2}}{{\gamma }_{2}+{\gamma }_{l}+2\frac{{\omega }_{0}-\omega }{{\omega }_{0}}i}$$

Although $g$ is intrinsically a complex function of operating frequency $\omega$, the setup of a PT-symmetric WPT system with a current-to-voltage feedback loop forces $g$ to be a real number. Thus, we can obtain the steady state by solving ${{{\rm{Im}}}}\left(g\right)=0$. The behavior of steady-state parameters (frequency ${\omega }_{{{{\rm{ss}}}}}$ and gain ${g}_{{{{\rm{ss}}}}}$) varies significantly depending on the interplay of the coupling strength and the receiver loss rates. Specifically, in the broken PT regime (where $k\le {\gamma }_{2}+{\gamma }_{l}$), the system supports a single natural resonant frequency ${\omega }_{0}$. Conversely, in the exact PT regime (where $k > {\gamma }_{2}+{\gamma }_{l}$), the system exhibits two extra splitting modes with frequency difference, expressed as ${\omega }_{{{{\rm{diff}}}}}=\pm \frac{{\omega }_{0}}{2}\sqrt{{k}^{2}-{\left({\gamma }_{2}+{\gamma }_{l}\right)}^{2}}$. The frequency spectrum of the PT-symmetric system as a function of ${\gamma }_{l}$ is shown in Fig. 1b. It is observable that in the exact PT region, the system supports two splitting resonant frequencies. As ${\gamma }_{l}$ increases to ${\gamma }_{c}=k-{\gamma }_{2}$, the system reaches a critical state (i.e., the EP), where the splitting frequencies converge to the natural resonant frequency, resulting in a constant frequency line across the broken PT region.

With the knowledge of steady-state frequency, the corresponding steady-state gain ${g}_{{{{\rm{ss}}}}}$ can be derived as ${\gamma }_{1}+{k}^{2}/\left({\gamma }_{2}+{\gamma }_{l}\right)$ in the broken PT region and ${\gamma }_{1}+{\gamma }_{2}+{\gamma }_{l}$ in the exact PT region. In the general case, $g$ may possess an imaginary part, which represents the reactive power oscillating between the source and load that has no impact on the transferred active power or efficiency. Consequently, the WPT efficiency is defined as follows:

3 $$\eta =\frac{{{{\mathrm{Re}}}}\left(g\right)-{\gamma }_{1}}{{{{\mathrm{Re}}}}\left(g\right)}\times \frac{{\gamma }_{l}}{{\gamma }_{2}+{\gamma }_{l}}$$

In the steady state of a PT-symmetric WPT system, the value of $g$ is forced to be real and ${{\mathrm{Re}}}\left(g\right)={g}_{{{{\rm{ss}}}}}$ holds. It can be demonstrated that $\eta$ increases linearly with ${\gamma }_{l}$ in the exact PT region, indicating that EP is the maximum efficiency point, where the total receiver loss equals the coupling strength. By solving $\frac{\partial \eta }{\partial {\gamma }_{l}}=0$, we can get the optimal loss rate in the broken region. In summary, the load conditions for achieving maximum efficiency in the two PT regions are as follows:

4 $${\gamma }_{l,{{{\rm{opt}}}}}=\left\{\begin{array}{cc}\sqrt{{\gamma }_{2}^{2}+\frac{{\gamma }_{2}}{{\gamma }_{1}}{k}^{2}}, & {{{\rm{Broken\; PT}}}}\\ k-{\gamma }_{2}, & {{{\rm{Exact\; PT}}}}\end{array}\right.$$

To unify the two PT regions, we adopt two assumptions without losing generality. First, when the transmitter and receiver coils are wound with the same material, they possess the same quality factor, i.e. ${\gamma }_{1}={\gamma }_{2}$, regardless of their distinct dimensions. Second, $k\gg {\gamma }_{2}$ holds for a WPT system with high quality factors at the EP. Then it can be deduced from Eq. (4) that ${\gamma }_{l,{{{\rm{broken}}}}}\approx {\gamma }_{l,{{{\rm{exact}}}}}$. In other word, ${\gamma }_{l,{{{\rm{opt}}}}}\approx {\gamma }_{c}$ holds for both the broken PT region and the exact PT region. At the EP, two splitting eigenfrequencies are degenerate and converge to ${\omega }_{0}$. Combining the optimal loss and frequency conditions, we can conclude that the EP is the global maximum efficiency point. Substituting ${{\mathrm{Re}}}\left(g\right)=k+{\gamma }_{1}$ and ${\gamma }_{l}={\gamma }_{c}$ into Eq. (3), we can derive the maximum transfer efficiency corresponding to the EP:

5 $${\eta }_{\max }=\frac{k-{\gamma }_{2}}{k+{\gamma }_{1}}$$

Notably, the numerator in Eq. (5) is just ${\gamma }_{c}$, reflecting the threshold at which two eigenfrequencies coalesce. It is evident from Eq. (5) that maximum transfer efficiency increases with the enhancement of coupling strength provided that the optimal loss condition is satisfied. The transmission efficiency is graphed with respect to ${\gamma }_{l}$ and $k$ ranging from 0 to 0.1 as depicted in Fig. 1c based on Eq. (3). The black dashed line in the diagram, formed by EPs, divides the system into two distinct PT regions. A detailed slice view of Fig. 1c is presented in Fig. 1d, showcasing efficiency curves corresponding to several discrete coupling strengths. Noted that the colored dots in both figures correspond to the same set of EPs. It is shown that the transmission efficiency increases with the rise of ${\gamma }_{l}$, peaking at the EP, and then diminishes with further increments. While the above analysis is conducted by using the coupled-mode theory, the electric circuit theory-based analysis presents coherent results (see Supplementary Information S1).

The gain element with nonlinear saturation characteristics is the core component for achieving PT symmetry in WPT circuits. As highlighted in Eq. (3), the WPT efficiency is contingent upon the steady-state value of $g$. Through our analysis, we uncover an implicit symmetry-breaking phase transition depicted in the modulus of gain ($\left|g\right|$) as shown in Fig. 1e. Specifically, when ${\gamma }_{l} > {\gamma }_{c}$, $\left|g\right|$ initially decreases before subsequently rising with increasing frequency, reaching its minimum at ${\omega }_{0}$. Conversely, when ${\gamma }_{l} < {\gamma }_{c}$, $\left|g\right|$ displays a distinctive “W” shape characterized by two minima. Notably, the bifurcation frequencies do not align precisely with the minima of $\left|g\right|$ but exhibit slight offsets, as they are determined solely by the imaginary part of $g$ as illustrated in Fig. 1f. The symmetry-breaking phase transition depicted in the plot of $g(\omega )$ serves as the intrinsic driving force of the peak transmission efficiency observed at the EP.

### Achieving maximum efficiency via virtual loss transformation

**Fig. 2:**
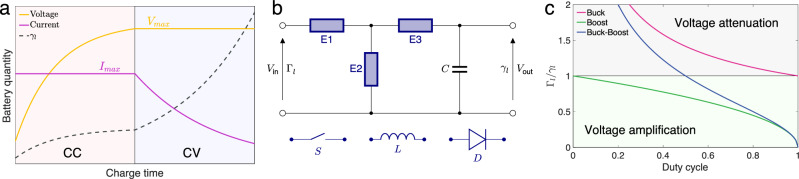
Mechanism of virtual loss transformation. **a** Voltage, current and equivalent loss curves in a typical charging process of lithium-ion batteries; CC constant current, CV constant voltage. **b** A general circuit model of three dc-dc converters for loss transformation; E1, E2, and E3 represent configurable circuit elements; S switch, L inductor, D diode, C dc filter capacitor. Buck converter is configured by S-D-L with respect to E1-E2-E3; boost and buck-boost converters are configured by L-S-D and S-L-D, respectively. **c** Virtual loss transformation ratios of three switch-mode dc-dc converters in continuous current mode; ${\varGamma }_{l}$ represents the virtual loss.

From the above analysis, it is desirable that a PT-symmetric WPT system operates at the EP for efficient power transfer, which requires setting the load loss rate at its optimal value. However, the load loss rate in practical circuits is rarely constant. For instance, consider the charging process of a lithium-ion battery, which typically adopts the constant current-constant voltage (CC-CV) mode^40^. In this mode, charging initially proceeds with the maximum current until the battery voltage reaches a peak value, followed by constant voltage charging^41^ (Fig. 2a). During the CC phase, the terminal voltage of the battery continually increases, whereas in the CV phase, the charging current gradually decreases, indicating a continual rise in the battery’s equivalent loss rate throughout the charging process. The variable loss characteristic of lithium-ion batteries poses challenges for the efficient operation of WPT systems, a common issue in scenarios such as wireless charging of smartphones and electric vehicles.

Directly changing the charging characteristics ${\gamma }_{l}$ of a battery is difficult, so an alternative approach is to adjust its virtual (equivalent) loss rate ${\varGamma }_{l}$ instead. One way to modify the virtual loss rate is by introducing an intermediary relay coil and then adjusting the coupling strength between this relay coil and the receiving coil^42^. This adjustment typically involves adding a mechanical element to regulate the separation distance between the coils. A more adaptable and accurate approach involves utilizing a dc-dc converter to fine-tune the virtual loss rate, ultimately enhancing the transfer efficiency. Although battery charging is ultimately a dc process, a switch‐mode converter inherently operates via high‐frequency switching and thus presents an effective ac impedance to the WPT system. The inductor, capacitor, and switching device create pulsating currents at the switching frequency (e.g., tens to hundreds of kilohertz), which are then averaged and filtered to produce a quasi‐dc output^43^. This perspective allows us to model the power transfer dynamics consistently with ac circuit concepts. The three commonly employed types of switch-mode dc-dc converters share a comparable circuit topology (Fig. 2b), comprising a power switch with duty cycle modulation, an inductor for storing and releasing energy, a diode for creating a current pathway, and a capacitor for filtering voltage ripples.

Proper arrangement of three circuit elements (E1, E2, and E3) results in the configuration of buck, boost, and buck-boost converters, each exhibiting distinct virtual loss transformation ratio ${\varGamma }_{l}/{\gamma }_{l}$ as a function of the duty cycle (derived in detail in Supplementary Information S2), as illustrated in Fig. 2c. The buck converter is designed for reducing dc voltage, while the boost converter is intended for increasing it. In contrast, the buck-boost converter offers the flexibility to both amplify and diminish voltage by adjusting the duty cycle of the power switch. Following the principle of power conservation before and after transformation, the voltage amplification by a dc-dc converter correlates with a reduction in virtual loss, and conversely, a decrease in voltage results in an increase in virtual loss. Therefore, integrating the dc-dc converter before the battery stage in the WPT system can help maintain an optimal virtual loss rate.

**Fig. 3:**
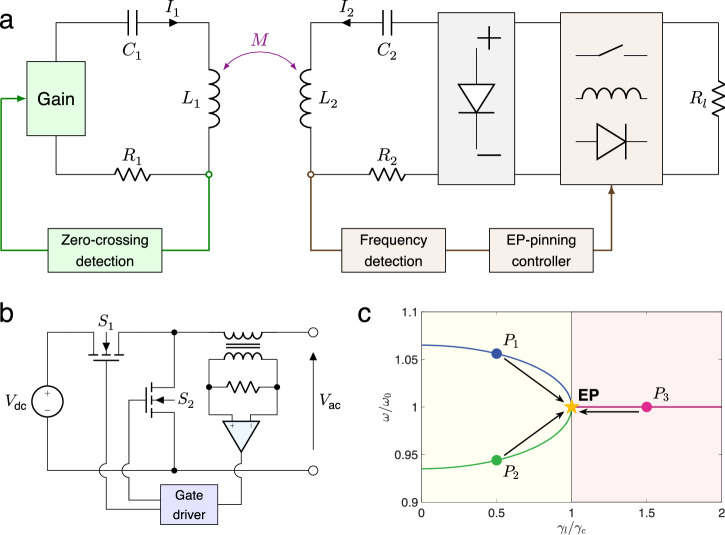
Design of the EP-pinning control system for maximum efficiency tracking. **a** Electric circuit model for the WPT system with a nonlinear gain element on the transmitter side and an EP-based controller on the receiver side. **b** Half-bridge inverter-based gain circuit with current-feedback self-oscillation. **c** Frequency shifting map of a PT-symmetric WPT system for achieving maximum efficiency by virtual loss transformation.

Traditional maximum efficiency tracking schemes for WPT typically employ a voltage source operating at a constant frequency. These control schemes involve computing ${\gamma }_{l,{{{\rm{opt}}}}}$ using system parameters (${\gamma }_{1},{\gamma }_{2}$, and $k$), and subsequently adjusting the duty cycle of the dc-dc converter according to the actual load loss^38,39^. This approach, however, necessitates precise and real-time measurement of multiple system parameters, which adds complexity to the circuit design. In response, we develop a maximum efficiency tracking system (Fig. 3a) based on EPs, utilizing a nonlinear saturation gain element as the energy injection source, along with a frequency-based linear controller to regulate the duty cycle of the dc-dc converter. Here, the WPT system consists of a pair of coupled inductors ${L}_{{{\mathrm{1,2}}}}$ with mutual inductance $M$, parasitic resistors ${R}_{{{\mathrm{1,2}}}}$, series compensating capacitors ${C}_{{{\mathrm{1,2}}}}$ and a load resistor ${R}_{l}$. In previous research, operational amplifiers^34^ and class-E power amplifiers^35^ were used to design the nonlinear gain elements, but these were not ideal for high-power operations. In this study, we opt for a half-bridge inverter with current feedback (refer to Fig. 3b), an extensively adopted component in power electronics, to serve as the nonlinear gain^37^. Specifically, the current at the transmitter side is converted into a low-power signal via a current transformer, and then fed into a voltage zero-crossing comparator to obtain the control signal of the gate driver. This self-oscillating circuit maintains a 180-degree phase difference between output current and voltage, effectively acting as a negative resistance component and automatically adjusting its operating frequency based on circuit loss and coupling conditions.

In the absence of extra control mechanisms, a PT-symmetric WPT system operates at one of the bifurcation frequencies within the exact PT region instead of at ${\omega }_{0}$, primarily due to the saturation nonlinearity of the gain. Conversely, in the broken PT region, it operates at ${\omega }_{0}$. Notably, operating at ${\omega }_{0}$ within the exact PT region results in a larger gain rate for WPT systems compared to two bifurcation frequencies, thereby achieving higher transmission efficiency. This indicates that the robustness of PT-symmetric systems comes at the cost of reduced transmission efficiency. According to the spectral graph (Fig. 3c), within the exact PT region, the operating frequency shifts along the bifurcation curve, converging precisely to ${\omega }_{0}$ when ${\gamma }_{l}={\gamma }_{c}$ (i.e., at the EP), demonstrating a direct correspondence between transmission efficiency and frequency. In the context of the broken PT region, where the system consistently operates at ${\omega }_{0}$, inferring the system’s transmission efficiency solely based on self-oscillating frequency proves challenging. Nonetheless, setting the target operating frequency close to ${\omega }_{0}$ at either ${\omega }_{-}$ or ${\omega }_{+}$ allows for an assessment of whether the system attains peak efficiency across all regions. Specifically, if the system’s present state is either $\omega > {\omega }_{+}$ (${P}_{1}$) or $\omega < {\omega }_{-}$ (${P}_{2}$), then ${\gamma }_{l}$ should be increased; otherwise ${\omega }_{-} < \omega < {\omega }_{+}$ (${P}_{3}$), it should be decreased. Given that the loss transformation ratio of the dc-dc converter is monotonic with respect to the duty cycle, a proportional-integral (PI) controller can be utilized to effectively implement this control strategy.

**Fig. 4:**
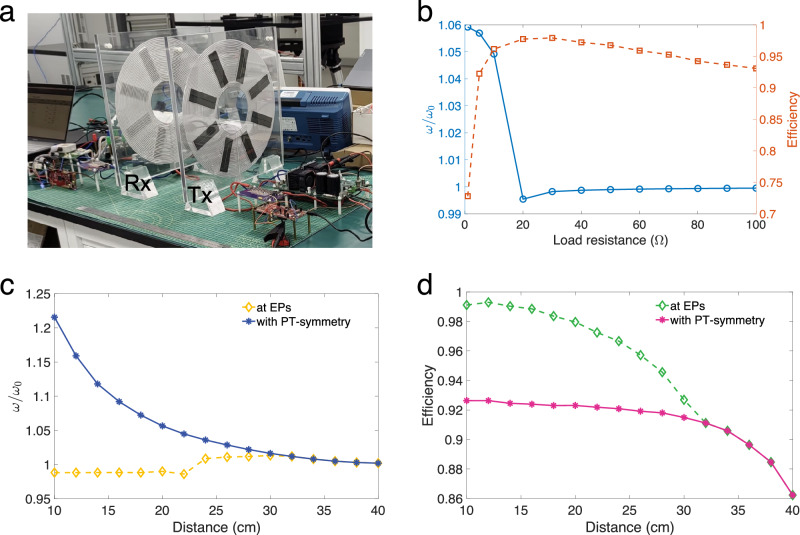
Experimental verification of the EP-based WPT. **a** Photograph of our experiment platform. Eight rectangular ferrite blocks are placed across the spiral grooves of each coil. **b**, Measured self-oscillation frequency (blue line and left *y* axis) and corresponding WPT efficiency (red dashed line and right *y* axis) as a function of load resistance, where the transfer distance is fixed at 20 cm. **c**, **d** Measurements of frequency (**c**) and efficiency (**d**) at different lateral separation distance between transmitter and receiver coils from 10 to 40 cm in 2-cm increments for a conventional PT symmetry-based scheme (solid line) and the proposed EP-based scheme (dashed line), given a constant load resistance of 5 Ω.

We constructed an experimental prototype (Fig. 4a) to verify the correlation between WPT efficiency and EPs and to implement maximum efficiency tracking, in which a buck-type dc-dc converter is used for virtual loss transformation. Notably, ferrite blocks are equipped with each coil, which helps guide the magnetic flux, reduce leakage, and boost coupling. Initially, a fixed transmission distance was set, and a range of load resistances from 1 to 100 Ω was connected to the circuit using an electronic load device. We calculated self-oscillation frequency and transmission efficiency based on voltage and current signals sampled by an oscilloscope (Fig. 4b). We continuously sampled the steady-state waveform at a sampling rate of 62.5 MHz for 4 ms and averaged the data to reduce measurement errors. To validate the effectiveness of the proposed EP-pinning strategy, we focus on evaluating the transfer efficiency of the wireless power link. The power conversion efficiency of the primary-side inverter and the secondary-side rectifier can be further optimized through advanced techniques such as soft-switching and active rectification. It is observed that the operating frequency of the PT-symmetric system decreases along the high bifurcation branch as the load resistance increases, converging near ${\omega }_{0}$ at a resistance of 20 Ω, corresponding to the EP. As the load resistance increased further, there is a slight rise in frequency due to high-order harmonics produced by the half-bridge inverter. Furthermore, the transfer efficiency displays an initial ascent followed by a descent with the escalation of load resistance, reaching its pinnacle around 20 Ω and proving that the maximum efficiency is achieved at the EP.

Following this, we conducted a series of tests to evaluate the performance of the EP-based virtual loss transformation controller across various transmission distances, contrasting its efficacy with the conventional PT-symmetric WPT scheme. The natural resonant frequency of our prototype WPT system is designed around 85 kHz, which is a widely adopted value, especially for wireless charging of electric vehicles. Here, we set the target frequency at either ${\omega }_{-}={\omega }_{0}-1\,{{{\rm{kHz}}}}$ or ${\omega }_{+}={\omega }_{0}+1\,{{{\rm{kHz}}}}$, which allows us to reduce the complexity of frequency identification without significantly impacting the resolution of EP-pinning. As illustrated in Fig. 4c, the self-oscillation frequency at EPs remains near ${\omega }_{0}$, whereas the frequency associated with PT symmetry decreases with transmission distance before eventually converging near ${\omega }_{0}$. Moreover, Fig. 4d showcases that the transmission efficiency at EPs consistently outperforms that of PT symmetry when the transmission distance varies between 10 and 30 cm. As the distance exceeds 30 cm, the self-oscillation frequency and transmission efficiency of the two systems coincide because the optimal loss rate falls below the given load loss, and the regulatory capacity of the buck converter reaches its limit. It’s noteworthy that when the load resistance is relatively large, the secondary compensation network should change from a series to a parallel topology^34^, ensuring the loss rate remains within the adjustable range. Overall, the transmission efficiency at EPs exhibits a decline with extended transfer distances as predicted by Eq. (5), while the efficiency with PT symmetry remains nearly constant at short distances, underscoring the trade-off between system robustness and diminished transfer efficiency. Also noteworthy is the observation that a reduction in transfer distance from 12 to 10 cm results in a decline in transfer efficiency at EPs, likely due to the magnified self-inductance induced by the ferrite surrounding the transmitter and receiver coils at close distances, causing a shift in the natural resonant frequency of the WPT system. This short-range behavior suggests that ferrite materials should be used with caution in strongly coupled WPT systems, or alternatively, multi-turn coils with a high quality factor should be employed to enhance coupling.

## Conclusions

In our investigation into the efficiency of PT-symmetric WPT systems, we establish theoretically that the transmission efficiency peaks at the EP, where the total loss of the receiver matches the coupling coefficient. Leveraging the frequency characteristics of the PT-symmetric system, we design an EP-based maximum efficiency tracking strategy that automatically adjusts the virtual loss of the load using a switch-mode dc-dc converter. This innovative strategy presents a streamlined circuit configuration compared to conventional methods, as it solely necessitates the monitoring of the self-oscillation frequency. Our system achieves frequency-stable operation, circumventing the generation of detrimental interference associated with variable frequency systems and simplifying the design of the filtering circuit. The empirical frequency and efficiency data obtained from our experimental prototype corroborate the theoretical projections. Our discovery paves the way for the effective operation of PT-symmetric WPT systems, showcasing substantial promise for deployment in free-moving IoT devices or high-power dynamic wireless charging of electric vehicles.

To address potential real-world deployment challenges, we note that the cost, safety, regulatory compliance, and scalability of PT-symmetric WPT systems should be carefully considered. The specialized materials (e.g., ferrite blocks, Litz wire) and power electronics (e.g., inverters, rectifiers, and dc-dc converters) used in our prototype may increase initial manufacturing expenses. However, mass production and material optimization can reduce these overheads. Safety and regulatory aspects revolve primarily around electromagnetic compatibility and exposure limits. Existing standards such as the SAE J2954 (for wireless electric vehicle charging) and CISPR guidelines impose strict limits on electromagnetic emissions to ensure minimal interference with other devices and to guarantee user safety. In practice, the coupling coils and resonant structures would require careful shielding and compliance testing to meet these standards. Scalability to higher power levels is achievable by proportionally increasing coil dimensions, ferrite arrangements, and the ratings of associated power electronics.

## Methods

### Coupled-mode parameters for the PT-symmetric WPT system

The coupled-mode model in Eq. (1) corresponds to the electric circuit model in Fig. 3a as follows: ${\omega }_{0}=\frac{1}{\sqrt{{L}_{1}{C}_{1}}}\approx \frac{1}{\sqrt{{L}_{2}{C}_{2}}}$ is the natural resonant frequency of two resonators, which are tuned to be approximately identical; $k=\frac{M}{\sqrt{{L}_{1}{L}_{2}}}$ is the coupling coefficient between two resonators; ${\gamma }_{1}=\frac{{R}_{1}}{{\omega }_{0}{L}_{1}}$, ${\gamma }_{2}=\frac{{R}_{2}}{{\omega }_{0}{L}_{2}}$, and ${\gamma }_{l}=\frac{{R}_{l}}{{\omega }_{0}{L}_{2}}$ are the loss rates of the series-connected transmitter resonator, receiver resonator and resistive load, respectively.

### Experiment set-up

Our experimental prototype comprised a dc voltage source, a half-bridge gallium nitride inverter (GSP65RxxHB-EVB), two series-connected compensating capacitors, a pair of ferrite-enhanced circular coils, a full-wave rectifier, a buck converter, a programmable electric load, and two types of microcontrollers (TMS320F28062 and TMS320F28379). The embedded digital comparator of TMS320F28379 was used for zero-crossing detection, supporting transmitter-side self-oscillation feedback and receiver-side frequency detection. Meanwhile, the TMS320F28062 microcontroller functions as a PI controller according to the frequency information obtained from its embedded capture module. Instrumentation utilized for measurements included a digital oscilloscope (TeK-MSO46), an LCR meter (TH2827), and a multimeter. To secure the spiral-wound circular coil and the accompanying ferrite block, a specially designed coil tray with dimensions of 40 × 40 × 1 cm^3^ featuring grooves was custom-made. The tray incorporated a segmented spiral track on the front, measuring 3×3 mm^2^, intended for winding the Litz wire, with 1 mm spacing between the spiral grooves. On the back of the tray, eight rectangular grooves were crafted to accommodate the ferrite blocks. The Litz wire used in this experiment is specified as $0.1\,{{{\rm{mm}}}}\times 400$ turn, with an outer diameter of $2.8\,{{{\rm{mm}}}}$ and a maximum current withstand capacity of $15.7\,{\mbox{A}}$. The ferrite material employed was PC95, measuring 90 × 16 × 5 mm^3^. The transmitter and receiver coils manufactured possess a self-inductance of 273 μH and an internal resistance of 0.21 $\Omega$. Additionally, two compensating capacitors of 12.63 nF were serially connected with the coils to tune the natural resonant frequency to approximately 85 kHz. To control variables, we fixed the input voltage at 30 V to examine the output characteristics under different coupling strengths and load resistance conditions.

## Supplementary information

Supplementary Information

## Data Availability

The data that support the findings of this study are available from the corresponding authors upon reasonable request.
