# Supplementary material for: Maximizing wireless power transfer efficiency at exceptional points
Source: Commun Eng. 2025 Jun 10;4:105. doi: 10.1038/s44172-025-00445-y (PMC12152173; doi:10.1038/s44172-025-00445-y)
Supplement: Supplementary file 1 — Supplementary Information [file 44172_2025_445_MOESM1_ESM.pdf]

Maximizing wireless power transfer efficiency at exceptional points: Supplementary information

Wei-Kang Hu[1,2], Bowang Zhang[2], Youhao Hu[2], Haoxiang Li[3]✉, Wei Han[2]✉

[1]Division of Emerging Interdisciplinary Areas, The Hong Kong University of Science and Technology, Hong Kong SAR, China. [2]Sustainable Energy and Environment Thrust, The Hong Kong University of Science and Technology (Guangzhou), Guangzhou, China. [3]Advanced Materials Thrust, The Hong Kong University of Science and Technology (Guangzhou), Guangzhou, China. ✉e-mail: haoxiangli@hkust-gz.edu.cn; weihan@hkust-gz.edu.cn

S1. EP and WPT efficiency: circuit theory-based analysis

Using coupled-mode theory (CMT) offers several advantages: it enhances interdisciplinary applicability by providing a unified modeling approach for PT-symmetric systems, simplifies the derivation process with a lower system order and intuitive physical interpretation, and allows broader applicability to various WPT topologies under PT symmetry conditions. However, electrical circuit theory (ECT) provides higher modeling accuracy for electronic systems. Therefore, while CMT is used in the main text, ECT is employed in the following to offer a more precise modeling example for series-series topology. Notably, other compensation topologies, such as parallel-parallel[1] and series-parallel[2], can also be configured within the framework of PT symmetry for WPT systems.

S1.1 Derivation of resonant frequency

A nonlinear PT-symmetric WPT system, as shown in Supplementary Figure 1, is powered by a negative resistor $-R_g$, whose voltage and current have a phase difference of $180°$. The output of a negative resistor features the zero-phase-angle (ZPA) characteristic, which indicates that the WPT system self-oscillates at one of the ZPA resonant frequencies.

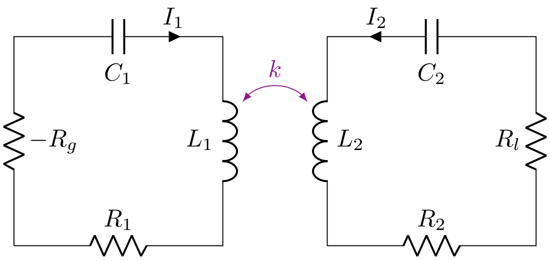

Supplementary Figure 1 | Circuit model of a WPT system with series-series compensation.

According to Kirchhoff's law, we can describe the circuit illustrated in Supplementary Figure 1 by the following equation

$$\begin{bmatrix} \frac{R_1 - R_g}{L_1} + j\left(\omega - \frac{1}{\omega L_1 C_1}\right) & j\omega k\sqrt{\frac{L_2}{L_1}} \\ j\omega k\sqrt{\frac{L_1}{L_2}} & \frac{R_2 + R_l}{L_2} + j\left(\omega - \frac{1}{\omega L_2 C_2}\right) \end{bmatrix} \begin{bmatrix} \dot{I}_1 \\ \dot{I}_2 \end{bmatrix} = 0 \quad \text{(S1)}$$

A necessary condition for PT-symmetry is that the two coupled resonators have the same natural resonant frequency

$$\omega_0 = \frac{1}{\sqrt{L_1 C_1}} = \frac{1}{\sqrt{L_2 C_2}}$$

Then Eq. (S1) can be rewritten as

$$\begin{bmatrix} \frac{R_1 - R_\mathrm{g}}{L_1} + j\left(\omega - \frac{\omega_0^2}{\omega}\right) & j\omega k\sqrt{\frac{L_2}{L_1}} \\ j\omega k\sqrt{\frac{L_1}{L_2}} & \frac{R_2 + R_l}{L_2} + j\left(\omega - \frac{\omega_0^2}{\omega}\right) \end{bmatrix} \begin{bmatrix} \dot{I}_1 \\ \dot{I}_2 \end{bmatrix} = 0 \tag{S2}$$

The sufficient and necessary condition for the homogeneous linear Eq. (S2) to have a non-zero solution is that the determinant of its coefficient matrix is zero, which gives

$$\left[\frac{R_1 - R_\mathrm{g}}{L_1} + j\left(\omega - \frac{\omega_0^2}{\omega}\right)\right]\left[\frac{R_2 + R_l}{L_2} + j\left(\omega - \frac{\omega_0^2}{\omega}\right)\right] + \omega^2 k^2 = 0$$

Separating the real and imaginary parts of the above equation, we can obtain

$$\frac{(R_1 - R_\mathrm{g})(R_2 + R_l)}{L_1 L_2} - \left(\omega - \frac{\omega_0^2}{\omega}\right)^2 + \omega^2 k^2 = 0 \tag{S3}$$

$$\left(\omega - \frac{\omega_0^2}{\omega}\right)\left(\frac{R_1 - R_\mathrm{g}}{L_1} + \frac{R_2 + R_l}{L_2}\right) = 0 \tag{S4}$$

Obviously, $\omega = \omega_0$ is a solution of Eq. (S4). Substituting $\omega = \omega_0$ into Eq. (S3) gives

$$R_\mathrm{g} = R_1 + \frac{\omega_0^2 k^2 L_1 L_2}{R_2 + R_l} \tag{S5}$$

For the case that $\omega \neq \omega_0$, Eq. (S4) supports another solution

$$R_\mathrm{g} = R_1 + \frac{L_1}{L_2}(R_2 + R_l) \tag{S6}$$

Substituting Eq. (S6) into equation Eq. (S3) gives two splitting resonant frequencies

$$\omega_{L,H} = \omega_0 \sqrt{\frac{2 - \gamma_s^2 \pm \sqrt{(2 - \gamma_s^2)^2 - 4(1 - k^2)}}{2(1 - k^2)}}$$

where

$$\gamma_s = \frac{R_2 + R_l}{\omega_0 L_2}$$

To ensure that the values of $\omega_{L,H}$ are real numbers, either $\gamma_s \leq \gamma_c$ or $k \geq k_c$ should be met, where

$$\gamma_c = \sqrt{2\left(1-\sqrt{1-k^2}\right)}$$

$$k_c = \sqrt{\gamma_s^2 - \frac{\gamma_s^4}{4}}$$

In summary, the ZPA resonant frequencies of the PT-symmetric WPT system is

$$\omega_{\text{ZPA}} = \begin{cases} \omega_0, & \gamma_s \geq 0 \\ \omega_{L,H}, & 0 \leq \gamma_s \leq \gamma_c \end{cases}$$

S1.2 Analysis of WPT efficiency

In the broken PT region, a PT-symmetric WPT system operates at the natural resonant frequency ω_0, at which the reflected resistance from the receiver side to the transmitter side is

$$R_{\text{ref}}(\omega = \omega_0) = \frac{\omega_0^2 k^2 L_1 L_2}{R_2 + R_l}$$

The WPT efficiency can be calculated by

$$\eta = \frac{R_{\text{ref}}}{R_1 + R_{\text{ref}}} \times \frac{R_l}{R_2 + R_l} = \frac{\omega_0^2 k^2 L_1 L_2 R_l}{[R_1(R_2 + R_l) + \omega_0^2 k^2 L_1 L_2](R_2 + R_l)}$$

Let the partial derivative of η with respect to R_l equal to zero $\partial\eta/\,\partial R_l = 0$, we get the optimal load condition for maximum efficiency transfer

$$R_{l,\text{opt}} = \sqrt{R_2^2 + \frac{R_2 L_2}{R_1 C_1} k^2}$$

and the corresponding WPT efficiency

$$\eta_{\max} = \frac{\frac{L_2}{C_1} k^2 \sqrt{R_2^2 + \frac{R_2 L_2}{R_1 C_1} k^2}}{\left[R_1\left(R_2 + \sqrt{R_2^2 + \frac{R_2 L_2}{R_1 C_1} k^2}\right) + \frac{L_2}{C_1} k^2\right]\left(R_2 + \sqrt{R_2^2 + \frac{R_2 L_2}{R_1 C_1} k^2}\right)}$$

By contrast, in the exact PT region, a PT-symmetric WPT system operates at one of two splitting resonant frequencies $\omega_{L,H}$, at which the reflected resistance is

$$R_{\text{ref}}(\omega = \omega_{L,H}) = \frac{L_1}{L_2}(R_2 + R_l)$$

The WPT efficiency at $\omega_{L,H}$ can be calculated as

$$\eta = \frac{R_{\text{ref}}}{R_1 + R_{\text{ref}}} \times \frac{R_l}{R_2 + R_l} = \frac{R_l}{\frac{L_2}{L_1} R_1 + R_2 + R_l}$$

from which we can see that η increases as R_l increases. Therefore, the maximum efficiency is achieved under the critical condition that $\gamma_s = \gamma_c$ ($k = k_c$), which corresponds to the EP. As such, the optimal load resistance is

$$R_{l,\text{opt}} = \omega_0 L_2 \sqrt{2\left(1-\sqrt{1-k^2}\right)} - R_2$$

and the corresponding WPT efficiency is

$$\eta_{\max} = \frac{\omega_0 L_2 \sqrt{2\left(1-\sqrt{1-k^2}\right)} - R_2}{\frac{L_2}{L_1} R_1 + \omega_0 L_2 \sqrt{2\left(1-\sqrt{1-k^2}\right)}}$$

The critical operating frequency ω_c for maximum efficiency is at the EP, which can be calculated as

$$\omega_c = \omega_{L,H}(k = k_c) = \frac{\omega_0}{\sqrt{1-\frac{\gamma_s^2}{2}}}$$

From the above analysis, we conclude that

$$R_{l,\text{opt}} = \begin{cases} \sqrt{R_2^2 + \frac{R_2 L_2}{R_1 C_1} k^2}, & \text{Broken PT} \\ \omega_0 L_2 \sqrt{2\left(1-\sqrt{1-k^2}\right)} - R_2, & \text{Exact PT} \end{cases}$$

$$\eta_{\max} = \begin{cases} \dfrac{\frac{L_2}{C_1} k^2 \sqrt{R_2^2 + \frac{R_2 L_2}{R_1 C_1} k^2}}{\left[R_1\left(R_2 + \sqrt{R_2^2 + \frac{R_2 L_2}{R_1 C_1} k^2}\right) + \frac{L_2}{C_1} k^2\right]\left(R_2 + \sqrt{R_2^2 + \frac{R_2 L_2}{R_1 C_1} k^2}\right)}, & \text{Broken PT} \\ \dfrac{\omega_0 L_2 \sqrt{2\left(1-\sqrt{1-k^2}\right)} - R_2}{\frac{L_2}{L_1} R_1 + \omega_0 L_2 \sqrt{2\left(1-\sqrt{1-k^2}\right)}}, & \text{Exact PT} \end{cases}$$

Notice that we have two sets of maximum efficiency conditions, and it is not obvious which one is optimal. To address this issue, we derive the optimal frequency for maximum efficiency in the following. The reflected resistance at any frequency can be calculated as

$$R_{\text{ref}} = \frac{\omega^2 k^2 L_1 L_2 (R_2 + R_l)}{(R_2 + R_l)^2 + \left(\omega L_2 - \frac{1}{\omega C_2}\right)^2}$$

The optimal frequency for maximum efficiency at any load condition can be derived by solving $\partial\eta / \partial\omega = 0$, which gives

$$\omega_{\mathrm{opt}} = \frac{1}{\sqrt{L_2C_2 - \frac{(R_2+R_l)^2C_2^2}{2}}}$$

Intriguingly, $\omega_{\mathrm{opt}} = \omega_c$ holds for PT-symmetric WPT systems, which indicates that ω_c (at the EP) is the optimal operating frequency for maximum efficiency transfer.

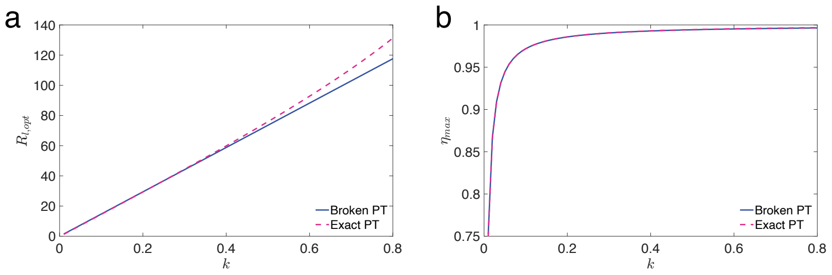

Supplementary Figure 2 | Optimal load resistance (**a**) and corresponding maximum efficiency (**b**) for broken PT and exact PT regions.

As can be seen from Supplementary Figure 2a, the optimal load resistance $R_{l,\mathrm{opt}}$ of two PT regions coincides when the coupling coefficient k is less than 0.4; $R_{l,\mathrm{opt}}$ in the exact PT region exceeds that in the broken PT region when k is further increases. Nevertheless, the maximum transmission efficiency $\eta_{\max}$ corresponding to two PT regions coincides for all coupling coefficients ranging from 0.01 to 0.8 as shown in Supplementary Figure 2b. In practical WPT systems, k is usually less than 0.3, under which the optimal loads and maximum efficiencies corresponding to two PT regions are approximately the same. Since the maximum transmission efficiency in the exact PT region is achieved at the EP. We can conclude that EP is the maximum efficiency point of the PT-symmetric WPT system.

S2. Loss transformation ratio of dc-dc converters

In this section, the loss transformation ratio of three dc-dc converters is derived. A dc-dc converter typically has two operational modes: continuous current mode (CCM) and discontinuous current mode (DCM). In CCM, the current flowing through the inductor I_L is always greater than 0 during a switching cycle. In contrast, I_L drops to 0 during the switch-off state in DCM. Generally, dc-dc converters are more efficient in CCM than in DCM.

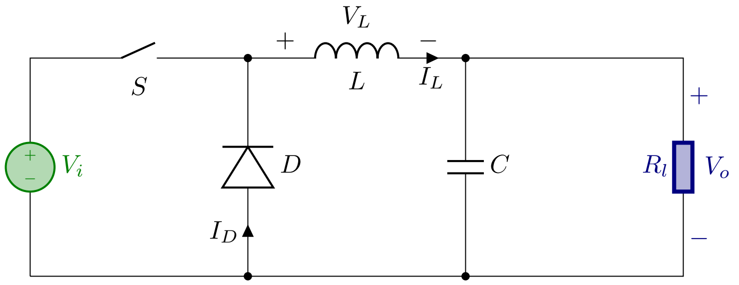

Supplementary Figure 3 | Circuit model of a buck converter.

At the boundary between CCM and DCM, I_L drops to 0 at the end of each switching cycle. This boundary condition is determined by a combination of inductance L, load resistance R_l, switching

period T, and duty cycle τ. Take the buck converter for example, as illustrated in Supplementary Figure 3. The minimum inductance for it to operate in CCM is

$$L_{\mathrm{min,buck}} = \frac{(1-\tau)R_l T}{2}$$

The voltage ratios of the buck converter in two working modes are

$$\frac{V_o}{V_i} = \begin{cases} \tau, & \mathrm{CCM} \\ \dfrac{2}{1+\sqrt{1+\dfrac{8L}{\tau^2 R_l T}}}, & \mathrm{DCM} \end{cases}$$

The equivalent resistance R_e can be calculated by the following expression

$$\frac{V_i^2}{R_e} = \frac{V_o^2}{R_l}$$

from which we get the loss transformation ratio for the buck converter

$$\xi_{\mathrm{buck}} = \frac{R_e}{R_l} = \begin{cases} \dfrac{1}{\tau^2}, & \mathrm{CCM} \\ \dfrac{\left(1+\sqrt{1+\dfrac{8L}{\tau^2 R_l T}}\right)^2}{4}, & \mathrm{DCM} \end{cases}$$

Similarly, the loss transformation ratios and minimum inductances for the boost converter and buck-boost converter can be derived as summarized in Supplementary Table 1.

Supplementary Table 1. Loss transformation ratios and minimum inductances of two dc-dc converters.

Type	ξ_{ccm}	ξ_{dcm}	Minimum inductance
Boost	$(1-\tau)^2$	$\dfrac{4}{\left(1+\sqrt{1+\dfrac{2\tau^2 R_l T}{L}}\right)^2}$	$\dfrac{\tau(1-\tau)^2 R_l T}{2}$
Buck-boost	$\dfrac{(1-\tau)^2}{\tau^2}$	$\dfrac{2L}{\tau^2 R_l T}$	$\dfrac{(1-\tau)^2 R_l T}{2}$

References

1. Assawaworrarit, S., Yu, X. & Fan, S. Robust wireless power transfer using a nonlinear parity-time-symmetric circuit. *Nature* **546**, 387–390 (2017).
2. Assawaworrarit, S. & Fan, S. Robust and efficient wireless power transfer using a switch-mode implementation of a nonlinear parity-time symmetric circuit. *Nat. Electron.* **3**, 273–279 (2020).
